# Data on comparison between FLEC and CLIMPAQ methods used for fast sorption measurements of VOCs on building materials

**DOI:** 10.1016/j.dib.2016.02.072

**Published:** 2016-03-07

**Authors:** Malak Rizk, Marie Verriele, Maxence Mendez, Nadège Blond, Sébastien Dusanter, Coralie Schoemaecker, Patrice Blondeau, Stéphane Le Calvé, Nadine Locoge

**Affiliations:** a Mines Douai, SAGE, F-59508 Douai, France; b Université de Lille, F-59000 Lille, France; c Université de Strasbourg/CNRS. Institut de Chimie et Procédés pour l’Energie, l’Environnement et la Santé (ICPEES), UMR 7515, F-67087 Strasbourg, France; d Université de Strasbourg/CNRS, Laboratoire Image, Ville, Environnement (LIVE), UMR 7362, F-67087 Strasbourg, France; e Laboratoire des Sciences de l’Ingénieur pour l’Environnement - LASIE FRE 3474 CNRS - Université de La Rochelle, La Rochelle, France; f Université de Lille 1, Laboratoire de Physico-chimie des Processus de Combustion et de l׳Atmosphère, F-59655 Villeneuve d׳Ascq, France

## Abstract

A test emission chamber called CLIMPAQ has been coupled to a chromatography analyzer GC to measure volatile organic compounds (VOC) concentration during a sorption experiments (Fast sorption measurements of VOCs on building materials: Part 2 – Comparison between FLEC and CLIMPAQ methods, (Rizk et al., In press) [1]). The equations used to calculate the mass transfer coefficient and the thickness of the boundary layer developed on the surface of a material are presented. In addition, the experimental profiles obtained using the CLIMPAQ chamber is also presented in the presence and the absence of a building material. Finally, the impact of chamber size on the obtained concentration profile using different chambers is shown using 3 types of chambers having different volumes, 1 m^3^, 30 m^3^ and a micro chamber of 40 mL.

**Specifications Table**

**Table:** 

Subject area	*Chemistry*
More specific subject area	*Indoor air quality*
Type of data	*Table, graph*
How data was acquired	*PTR-ToFMS (Kore technology)*
Data format	*Analyzed*
Experimental factors	*50±5% at 23±2 °C*
Experimental features	*Measurements of sorption parameters in a test chamber*
Data source location	*SAGE, Mines Douai (France)*
Data accessibility	*Data is within this article.*

**Value of the data**

• May be used to calculate the mass transfer coefficient in a CLIMPAQ test emission chamber.
• May be useful to compare the sink effect between the CLIMPAQ emission test chamber and other chambers used in literature.
• The role of the chamber size on the determination of sorption parameters during experimental works is not yet reported.

## 1 Data

Different mathematical models were used to determine the sorption parameters from experimental data taking into account different parameters such as the sink effect on empty chamber walls and the presence of a boundary-layer. For this, some equations are used to calculate the mass transfer coefficient, the thickness of the boundary layer developed on the surface of a material and the effect of the chamber volume.

## 2 Experimental design, materials and methods

To perform sorption experiments in a test emission chamber called CLIMPAQ, a blank experiment referred in the following as “No sink” is first performed using an empty chamber to evaluate the sink effect on the chamber walls. The same experiment is performed with the tested material. When experimental concentration profiles are obtained, they are analysed using a model is used to take into account the effect of the boundary layer [1]. According to [2] the calculation of the boundary layer thickness and the mass transfer coefficients is done using the following equations:

$$R_{eL}=\frac{UL}{\nu }$$

**Table:** 

**R**_**eL**_	the Reynolds number
**U**	the mean fluid velocity (parallel to the surface) outside of the boundary layer (m/s)
**L**	the length of the surface in the direction of the air flow (m)
**ν**	the kinematic viscosity of the air phase (m^2^/s)

$$S_{c}=\frac{\nu }{\alpha -air_{D}}$$

**Table:** 

**S**_**c**_	the Schmidt number
**α-air**_**D**_	the molecular diffusivity of the binary VOC a in the air (m^2^/s)

$$\overset{¯}{S_{hL}}=\frac{\overset{¯}{h_{m}}L}{\alpha -air_{D}}=0.664{\left(R_{eL}\right)}^{1/2}{\left(S_{c}\right)}^{1/3}\;\;Si\;R_{eL}\;<500,000$$

$$\overset{¯}{S_{hL}}=\frac{\overset{¯}{h_{m}}L}{\alpha -air_{D}}=0.037{\left(R_{eL}\right)}^{4/5}{\left(S_{c}\right)}^{1/3}\;SiR_{eL}\;>\;500,000\;et\;S_{c}\approx 1$$

**Table:** 

**S**_**hL**_	the Sherwood number
**h**_**mL**_	the average film mass transfer coefficient acting over the adsorbent surface (m/s)

$$\delta =\frac{L}{S_{hL}}$$

**Table:** 

**δ**	the thickness of the boundary layer (m)

**Fig. 1:**
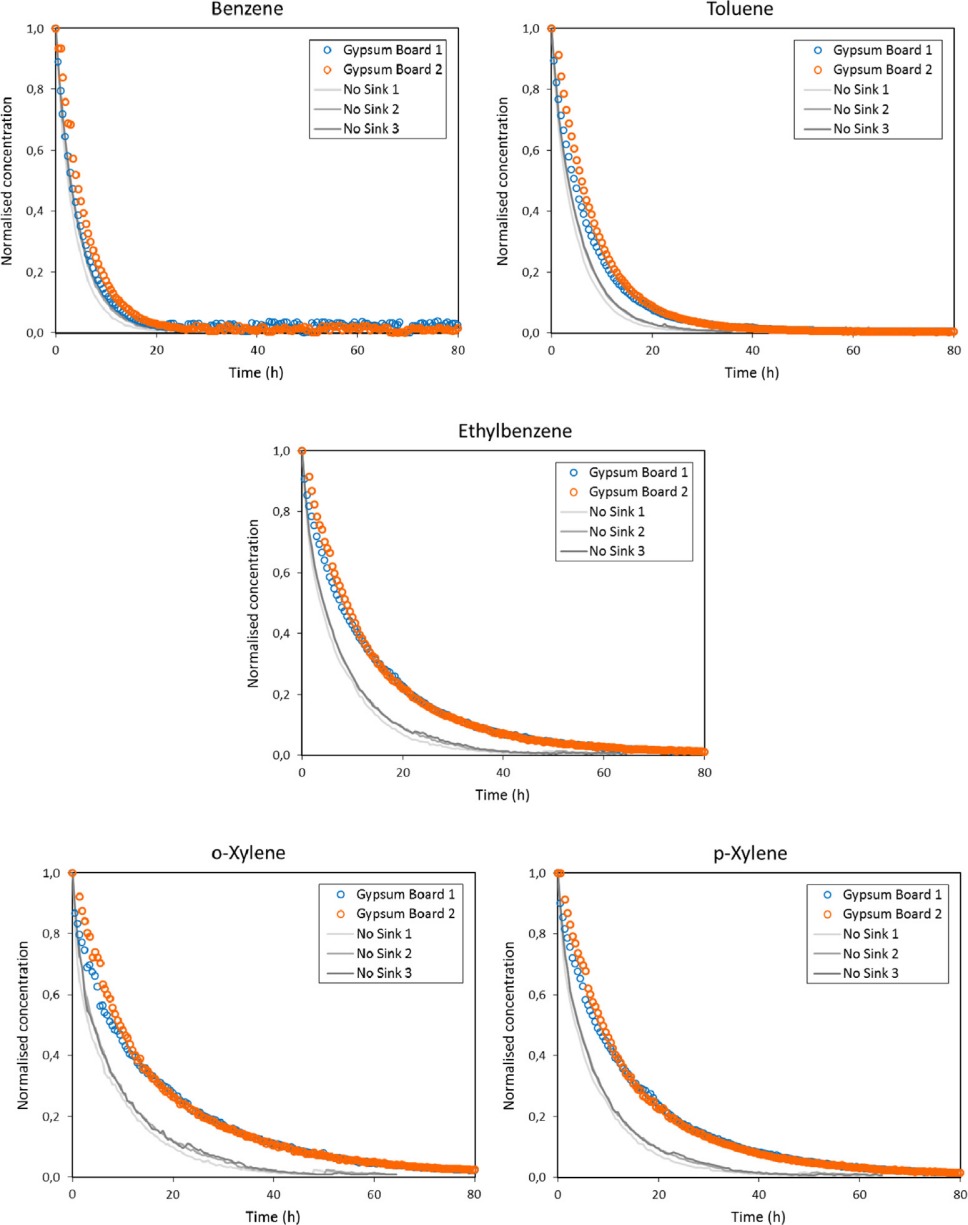
Comparison of the experimental profiles obtained for benzene, toluene, ethylbenzene and o-p/xylene with three experiments performed in an empty CLIMPAQ chamber (No Sink) and two experiments performed using the Gypsum board.

The experimental concentration profiles obtained for the three experiments performed in the empty emission test chamber (No Sink) show relatively good overlay as well as the two experiments performed with the gypsum board (Fig. 1).

**Fig. 2:**
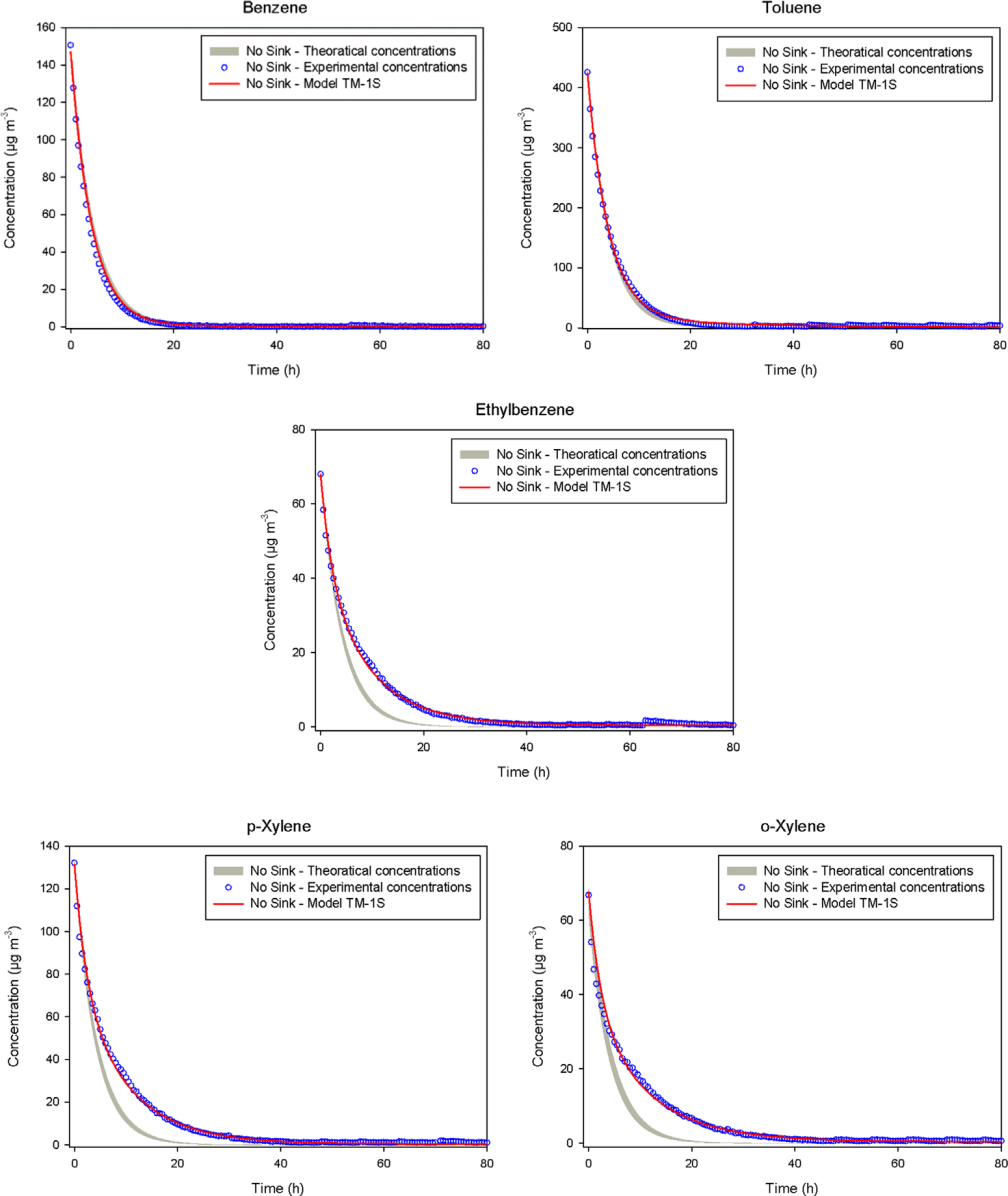
Analysis of the experimental No sink profiles obtained in empty CLIMPAQ, using the model TM-1S to extract sorption parameters of the benzene, toluene, ethylbenzene and o-p/xylene to the emission test chamber walls. The grey plot represents the theoretical concentration profile that should be obtained in empty chamber.

The sink effect on the chamber walls is investigated by calculating the theoretical concentrations that should be obtained for a blank experiment (No sink) under the conditions of this study and assuming negligible walls effects (Fig. 2). The equation used, accounts only for air exchange in the chamber and is $C(t)=C_{e}e^{-Nt}$ [3], with *C*(*t*) the concentration versus time (µg m^−3^), *C_e_* the equilibrium concentration reached at the end of the adsorption phase (µg m^−3^), and *N* the air exchange rate measured experimentally (h^−1^). The sorption parameters of VOCs on the chamber walls are determine using the Tichenor model called TM-1S according to M. Rizk [1]. Fig. 2 present the result of the model TM-1S which reproduces very well the experimental data.

The analysis of the impact of chamber size on the obtained sorption results is done using 3 types of chambers having different volumes, 1 m^3^, 30 m^3^and a micro chamber of 40 mL [4]. A set of desorption curves are simulated for each apparatus, using different couples of (*k_a_*; *k_d_*), but having the same *K_e_* ratio. A factor *α* varying between 0.01 and 100 is used to multiply both sorption parameters (*αk_a_*; *αk_d_*) as already presented in M. Rizk [1]. All the parameters used for the calculation are presented in Table 1.

**Table 1:** The parameters of the different chambers used.

**Parameters**	**Chamber of 1 m**^**3**^	**Chamber of 30 m**^**3**^	**Chamber of 40** **mL**
Volume (V; m^−^^3^)	1	30	4 10^−5^
Air exchange rate (Q; h^−1^)	0.01	0.01	300
Area of test piece (A; m^2^)	1	10	1.59 10^−3^
Loading factor (L; m^2^ m^−3^)	1	0.33	40

**Fig. 3:**
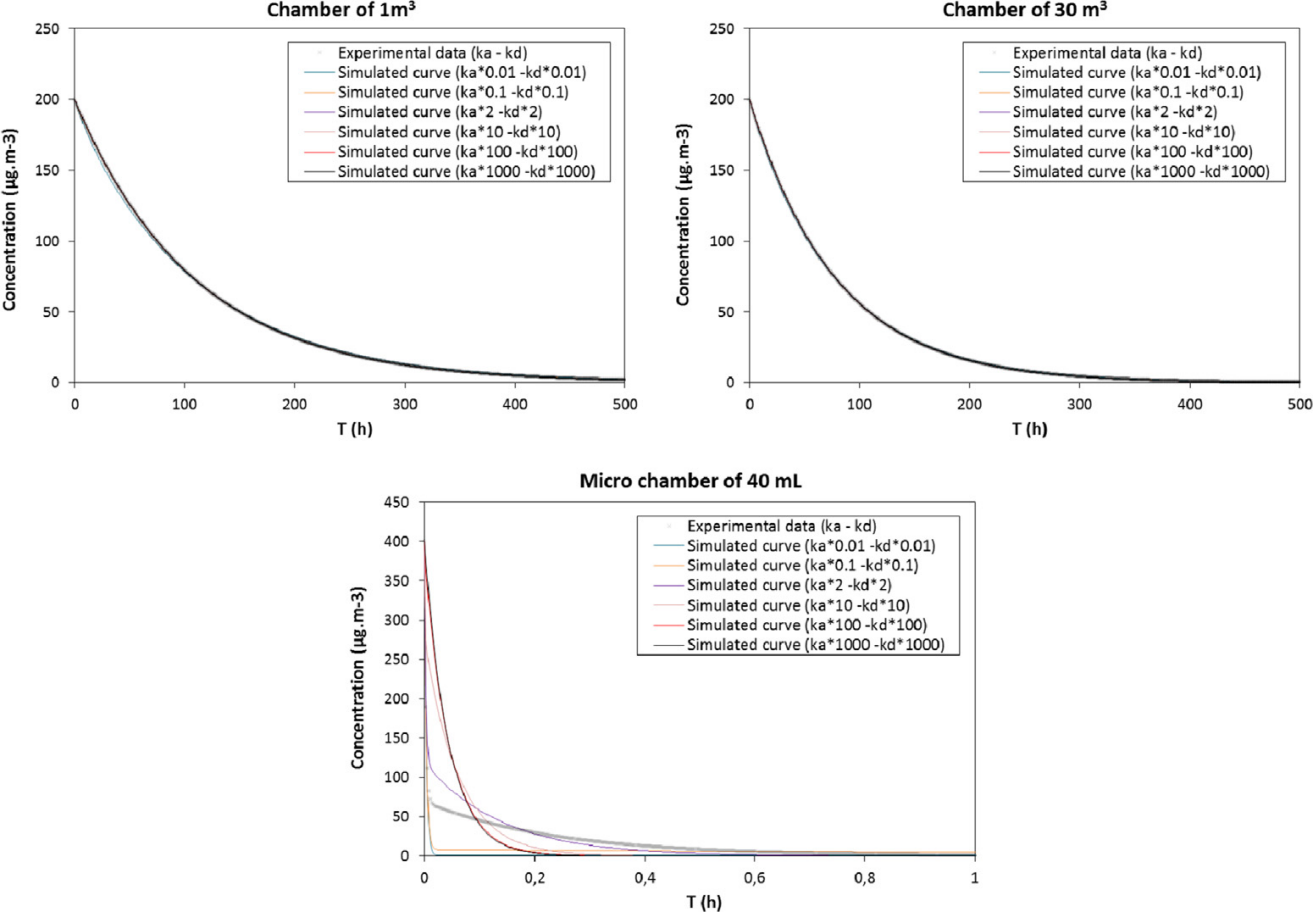
Results obtained for simulated curves for different factors α for toluene using three types of chambers having different volume.

Fig. 3 show the different desorption curves obtained using different couples of of (*k_a_*; *k_d_*), but having the same *K_e_* ratio, for the different chambers.
