# Supplementary material for: Data on comparison between FLEC and CLIMPAQ methods used for fast sorption measurements of VOCs on building materials
Source: Data Brief. 2016 Mar 7;7:518–23. doi: 10.1016/j.dib.2016.02.072 (PMC4796712; doi:10.1016/j.dib.2016.02.072)
Supplement: Supplementary file 1 — Supplementary material [file mmc1.docx]

**Conflict of interest**

Dear Editor

We do not have any conflict of interest in publishing the research work in Data in Brief.

Best Regards

Dr. Rizk

(on behalf of all authors)
